# Prevalence and factors associated with Acute Kidney Injury among children aged 6month- 12years passing dark urine admitted at Soroti Regional Referral Hospital: A cross-sectional study

**DOI:** 10.21203/rs.3.rs-2871679/v1

**Published:** 2023-05-08

**Authors:** Margaret Nakuya, Anthony Batte, Victor Musiime

**Affiliations:** Makerere University College of Health Sciences; Makerere University College of Health Sciences; Makerere University College of Health Sciences

**Keywords:** Children, cell-free haemoglobin, Acute kidney injury, Dark urine

## Abstract

**Introduction:**

Acute Kidney Injury (AKI) is associated with a high mortality yet survivors are at risk for Hypertension, chronic kidney disease, long term neurocognitive and behavioural problems. Early recognition of patients with possible AKI is crucial for better treatment outcome, hence a need for evidence to guide targeted screening of patients with a risk factor for AKI. We sought to determine the prevalence and factors associated with AKI among children passing dark urine since haemoglobinuria, which presents as dark urine is a known risk factor for AKI.

**Methodology:**

This was a cross sectional study conducted at Soroti Regional Referral Hospital, among children aged 6month to 12years, who presented with dark urine. Urine colour was assessed using the Hammer Smith colour urine chart, only children with urine colour grade ≥ 5 were recruited. Serum creatinine analysis was done on the day of admission, within 48 hours and at day 7 or discharge. AKI was defined as a ≥ 1.5-fold increase in serum creatininefrom the baseline. Bivariate and multivariate analysis was used to determine factors associates with AKI with p values <0.05 level of significance.

**Results:**

Between January 2022 – July 2022, we enrolled a total of 255 participantswith median age of 4.0 (IQR, 2.0-6.58) years. About two thirds of the participants were males 157 (61.6%) and majority 111 (43.5%) presented with grade 8 of the urine colour. The prevalence of AKI was 38% (95% CI 32.3% - 44.2%). The factors found to be associated with AKI were grade of the urine colour ≥ 9 (aOR, 3.120 (95% CI 1.34-6.78) and reduced urine output (aOR, 3.226 (95% CI 1.10-9.81)

**Conclusion;:**

The prevalence of AKI among children passing dark urine was high (38%). AKI was more likely to occur if the child was passing urine that is profoundly black and if there is history of reduced urine output. These findings reiterate the need for close monitoring of urine output of hospitalized children particularly those passing dark urine. Screening of those with profoundly black urine or reduced urine output should be done.

## Background

Acute Kidney Injury (AKI) is a sudden decline in the kidney function denoted by a change in serum creatinine or urine output (2). Haemoglobinuria, which is a known cause for AKI, reflects severe intravascular haemolysis and is associated passing dark urine. Normally, following haemolysis of red blood cells, free haemoglobin in the plasma dissociates into dimers that are immediately scavenged by plasma haptoglobin and cleared by hepatocytes. If the degree of intravascular hemolysis exceeds the scavenging capacity of plasma haptoglobin for cell-free haemoglobin (CFH), CFH gets filtered by the kidneys and appears in urine, giving it the characteristic dark colour (3, 4). Cell free haemoglobin can induce kidney injury via a number of mechanisms, including oxidative stress, cytotoxicity pathways, formation of intratubular casts and through proinflammatory effects (3, 5).Maintained intravascular haemolysis can produce chronic renal damage (6). Plewes et al studied the association between CFH and AKI and in her study, participants with a high CFH geometric mean were likely to develop AKI (3). Despite their sometimes similar visual appearances, the presence of haemoglobin on dipstick with absence of red blood cells on microscopic examination distinguishes haemoglobinuria from haematuria.

Haemoglobinuria, which presents as dark urine is one of the manifestations of severe malaria, primarily in falciparum infection (7). In the tropics it has been commonly associated with severe malaria (Blackwater fever) and Glucose-6-phosphate dehydrogenase (G6PD) deficiency (5). Other than Malaria, dark urine is observed during other hemolyzing pathologies including severe sepsis, haemoglobinopathies, drug reactions and intoxication, blood transfusion reactions, allergic reactions and herbal remedies (8).

Some studies have reported Haemoglobinuria associated AKI as high as 57.6% (8). In the multicenter Fluid Expansion As a Supportive Therapy (FEAST) trial that had 4sites in Uganda, 65.8% of the FEAST trial participants with haemoglobinuria showed evidence of renal impairment(9).

The factors shown to be associated with AKI among children passing dark urine from various studies include: older age (> 5years), male gender, underlying aetiology (malaria and sepsis), use of herbal medication, exposure to nephrotoxic drugs including NSAIDs and G6PD deficiency (8, 9).

## Methods

### Study design and setting

This was a cross section study conducted between January and July 2022. We recruited 255 children aged 6months-2years passing dark urine admitted at Soroti Regional Referral Hospital (SRRH) paediatric ward. SRRH is the main government referral facility for the mid-eastern region of Uganda (Teso-sub region). It’s located approximately 291 kilometres northeast of Kampala with a bed capacity of 300beds. The hospital offers specialist curative (medicine, pediatrics, surgery, maternal health) promotive, preventative, rehabilitative and research services to a population of 3.2 million with an annual overall admission of about 20000 patients.

The paediatric ward is a 64-bed capacity with an average annual admission of 7000 patients. The commonest causes of admission and deaths in the paediatric ward are: malaria, pneumonia, anaemia and neonatal conditions. On average, about 60 patients with dark urine are admitted in a month but this doubles towards the end of each rainy season (paediatric ward records). The hospital runs one paediatric follow up clinic that started as a sickle cell clinic but with time all other chronic illnesses were incorporated for follow up in the clinic, including Dark urine Syndrome (DUS). SRRH serves teso-sub region which is one of the regions with a high prevalence of dark urine as reported by previous studies (1, 9).

### Study procedures

The study team identified children (6month-12years) who presented with a history of passing dark urine in the preceding 7days as reported by the caregiver. The caregivers were shown the Harmmersmith urine colour chart and were asked to point to the colour of the child’s urine. Only children whose urine colour was grade 5 and above on the Hammersmith urine colour chart were regarded as having dark urine and were hence considered to have haemoglobinuria. Previous studies have reported an 80% agreement between parental report of dark urine with urine dipstick positive for haemoglobinuria(10). These were screened for eligibility and consent (and assent for those aged > 8years) was sought. Consecutive sampling was done until the sample size of 255 participants was achieved. A pretested questionnaire was administered to the caregivers of enrolled children to collect data that included; child’s demographics, symptoms about current illness, duration of the symptoms, episodes of passing dark urine since birth, number of previous blood transfusions related to passing dark urine, passed medical history, drug history including herbal medicine use, any chronic illnesses, family and social history. Each study participant under-went a physical examination including a general examination, systemic examination, anthropometry and blood pressure measurement, which was calculated as the mean of three independent measurements and Hypertension was defined a systolic and /or diastolic blood pressure > 95th percentile.

### Laboratory investigations

Urine and blood samples were obtained. Urine analysis included dipstick and microscopy.

Five (5) millilitres of venous blood were drawn. One (1ml) of blood was used for a complete blood count, malaria rapid diagnostic test (MRDT), blood smear for malaria parasites and a dry blood sample for Hb electrophoresis. The rest of the blood was used for analysis of serum creatinine. Within 48hours of admission and at day 7 of admission (or on the day of discharge whichever was earlier), other blood samples for serum creatinine were collected.

The samples were analyzed at Soroti Regional referral Hospital laboratory by the laboratory technician who has both the GCP and GCLP certificate. The hospital laboratory is accredited by the African Society for laboratory Medicine (ASLM) but also has certificate of practice from the Uganda’s Ministry of Health. The hospital laboratory uses a Cobas 311 make of the chemistry machine and serum creatinine was analysed by the Jaffe’s reaction method. For this study, AKI was defined based on the Kidney Disease: Improving Global Outcomes (KDIGO) guidelines as ≥ 1.5-fold increase in serum creatinine from the baseline within 7days (2). Each participant’s lowest measured creatinine of the three measurement was taken as the baseline serum creatinine for AKI determination as per the KDIGO guidelines (2).

Children from whom we were not able obtain the 3 serum creatinine measurements (n = 25), (such as those who died within 24hours of admission or referred to another facility), the Pottel height-independent GFR estimating equation was used to back calculate baseline creatinine, assuming a normal GFR of 120mL/min per 1.73m^2^. The Pottel-age based equation, was found to be the more accurate method with minimal bias when compared to the Schwartz height-based equation in a Ugandan study that was done to estimate the baseline creatinine in children with severe malaria (11). All participants received the standard of care as stated by the Uganda Ministry of health treatment guidelines. Any child found to have AKI was managed as per the local treatment guidelines which includes; Assessment of the fluid balance and administration of fluids if dehydrated or furosemide if overloaded, Daily monitoring of weight, Correction of electrolyte abnormalities, management of hypertension, ensure adequate nutrition, monitoring blood glucose and correction of hypoglycemia, discontinue nephrotoxic drugs, management of underlying illness such as administration of artesunate, counsel the caregivers on possibility of referral for dialysis if child’s condition worsens despite the above management. The children were followed up until discharge or day7 (whichever came earlier) to determine the vital status of either dead or alive. Upon discharge they were linked to the paediatric follow up clinic for regular reviews.

### Study variables

The primary outcome was AKI which was defined as ≥ 1.5-fold increase in serum creatinine from the baseline as per the Kidney Disease: Improving Global Outcomes (KDIGO) guidelines (2).

Independent variable included patient related factors like age, sex, weight and height, Underlying illness like Malaria, Sickle cell disease, sepsis and dehydration, treatment related factors like use of herbal medication, exposure to nephrotoxic drugs and severity of illness

### Statistical analysis

Data was analysed using STATA v15.0. Data was summarized descriptively using median and interquartile range (IQR) for continuous variables if skewed and means and standard deviation if normally distributed. Categorical variables were presented in terms of frequencies and proportions. The prevalence of AKI was calculated as a proportion of children with AKI among all those enrolled in the study. To determine factors associated with AKI among children passing dark urine, logistic regression was used to determine the factors independently associate with AKI and those with P value < 0.2 were included at multivariable model to assess the association between AKI among children passing dark urine and the independent variables. Adjusted odds ratios and corresponding 95% confidence intervals were reported at significance level *p* < 0.05.

## Results

**Figure 1:**
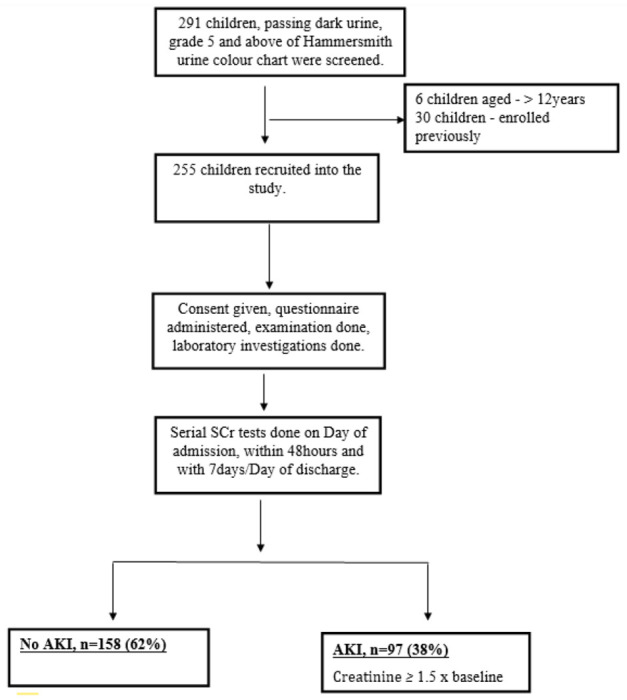
Study Profile

A total of 291 children, admitted with a history of passing dark urine were screened but 255 of these were enrolled (Figure 1). Thirty-six children were excluded (6 were above 12years of age, 30 were enrolled previously).

### Characteristics of study participants

A total of 255 participants were enrolled into the study (Table 1). Two thirds of all recruited participants were male n=157 (61.6%). Half of the participants were lying between 5-10years of age n=128 (50.2%). Majority 224 (87.8%) of the study participants had received pre-medication before presenting to SRRH which included antimalarials (84.4%), paracetamol (83.3%) and NSAIDs (diclofenac or Ibuprofen) (36.9%). Another 25.3% took herbal medication in this illness. Close to half of the study participants 104 (40.9%) this was their 2^nd^ – 5^th^ episode of passing dark urine. Majority of the participants (43.5%) presented with grade 8 on the harmer smith urine color chart. Table 1 and Table 2 below shows the baseline demographic and disease related characteristics.

### Prevalence of Acute Kidney Injury among children passing dark urine.

**Figure 2:**
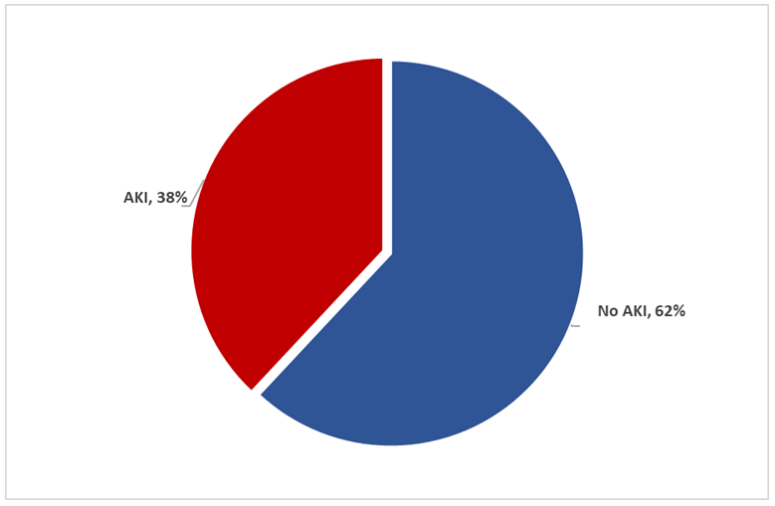
Prevalence of AKI among children aged 6months - 12years passing dark urine admitted at SRRH.

Of the 255 children in this study, 97 Had AKI, making the prevalence of AKI among children passing dark urine admitted at SRRH to be 38% (32.3%-44.2%) (Figure 2) Two thirds of the participants with AKI in this study were males. Half (50.2%) of the participants with AKI were ≥ 5years of age. Of the 97 participants that had AKI, 41/97(42.3%) had KDIGO stage I AKI, 27/97 (27.8%) had KIDIGO stage 2 while 29/255 (29.9%) had KDIGO stage 3.

### Factors associated with Acute Kidney injury.

To determine the factors associated with AKI among children passing dark urine, logistic regression analysis was used to test the association between AKI and the independent variables individually and any variable that achieved P value < 0.2 was found significant and was considered for multivariable model. Urine was profoundly black or coca cola coloured (≥ 9 colour grade on the urine colour chart), reduced urine output, early morning facial puffiness, convulsions, dehydration, altered level of consciousness and difficult in breathing were found to be significantly associated with AKI at bivariate analysis and these were considered in the multivariable model (Table 3). On addition, factors that are consistent with literature as being associated with AKI among children passing dark urine were also considered for multivariable model. These include age, male gender and underlying diagnosis of malaria. Table 3 below is showing bivariate and multivariable model.

## DISCUSSION

In this study, the prevalence of AKI among children passing dark urine was found to be 38% with 42.3% children having KDIGO stage I, 27.8% having stage II and 29.9% having stage III. The prevalence obtained in our study is high but studies have generally reported a high burden of AKI in developing countries especially because of the high burden of infectious diseases. The International Society of nephrology’s 0by25 initiative for acute kidney injury (zero preventable deaths by 2025) reported that AKI occurs in 13.3 million people annually and 85% of these occur in developing world (12). There has been a progressive increase in the prevalence of AKI among children in Uganda. In 2013, Imani et al reported a prevalence of 13.5% among children admitted with acute infections (13) and nearly a decade later there has been a 3-fold increase in the prevalence of AKI. Recently in 2022, Conroy et al reported a prevalence of AKI among children with a febrile illness to be at 49.5% (14). This high prevalence we found in our study is consistent with this observed trend.

The prevalence of AKI in our study was higher than that reported in a study done in Nigeria and another in Cameroon. In Nigeria, Waisu et al’s study entitled; Haemoglobinuria among children admitted with severe malaria attending tertiary care in Ibadan Nigeria, recruited 251 participants. Forty-eight (19.1%) children had haemoglobinuria and 6.25% of these developed acute renal failure (ARF) (15). This discrepancy could be explained by the fact that in this study they studied a much smaller sample size of only 48 participants compare to ours. Also, they also defined renal failure based on either the urine output of <12ml/kg/24hours or single plasma creatinine measurement which was different from how we defined AKI in our study(15).

In Cameroon, Kaptue et al reported that 21% of patients with AKI had haemoglobinuria as a risk factor(16). The lower prevalence reported in Cameroon could be attributable to the fact that in their study they enrolled children with various risk factors for AKI yet for us we only recruited children with dark urine.

Surprisingly, the prevalence obtained in our study was nearly half of that reported in the FEAST trial where 65.8% of the trial participants with BWF showed evidence of renal impairment. In that trail, they enrolled children with severe febrile illness and shock and they used BUN as a maker of kidney function as opposed to serial serum creatinine measurements which we used in our study. Various prevalences have also been reported by different authors elsewhere, and in most of these studies, the prevalence reported was higher than what we found in our study. In another study done in Nigeria for example, Asinobi et al found a prevalence of haemoglobinuria associated AKI to be at 57.6% for participants admitted in Paediatric Nephrology Unit and in this study only looked at KDIGO stage 3 AKI (8). In one study in Congo, of the 89 participants with ARF, 87 had Black Water Fever (17) while in Vietnam, they reported a prevalence of 42% (18). In both these studies, their sample size was lower than our study sample size.

In this current study, the factors that were significantly associated with AKI among children passing dark urine were grade of the urine colour and reduced urine output. Children who presented with urine that was of colour grade ≥ 9 on the HammerSmith urine colour chat were 3times more likely to develop AKI. Urine of colour grade 9 and above is profoundly black or Coca-Cola coloured and reflects the severity of intravascular hemolysis. Following massive intravascular haemolysis, Free hemoglobin is scavenged by plasma haptoglobin and if the scavenging capacity is exceeded, Cell Free Haemoglobin is then filtered by the glomeruli and will appear in urine, giving it the characteristic black urine. Exposure of the renal tubules to free haem will cause renal injury (19). It is therefore likely that children whose urine is profoundly black have higher concentration of cell free haemoglobin hence greater exposure of the renal tubules to the free haem. This could explain why those whose urine was grade ≥ 9 had higher were more likely to develop AKI. Plewes et al was able to associate CFH to AKI and in her study patients who had higher CFH geometric mean were more likely to develop AKI(3).

Another factor that was found to be significantly associated with AKI was reduced urine output. Children with dark urine who also had a reduced urine output were 3.4 times more likely to have AKI. Reduced urine output per se is one of the manifestations of AKI and it’s hence not surprising that children with reduced urine also had AKI. Reduced urine output in the background of dark urine is an important symptom that quickly points out those that may have AKI at presentation or may be used to monitor those likely to develop AKI during the duration of the episode of passing dark urine.

### Strength of the study

This study was carried out in a region with highest prevalence of dark urine in the country. Local studies in Uganda have described a high prevalence of BWF in Soroti.

Also, the method used to diagnose AKI is another strength for the study, which included serial measurement of serum creatinine (at admission, within 48 hours and at discharge or Day 7) as recommended by KDIGO guidelines in diagnosis of AKI as opposed to a single serum creatinine measurement which is susceptible to interference and may misclassify AKI.

### Study Limitation

We were not able to assess the underlying illness for febrile patients who had a negative malaria test. Blood cultures and G6PD tests were not done yet other studies have reported sepsis and G6PD deficiency as one of the underlying illnesses among children passing dark urine. Also, local studies done is Soroti have reported up to 15.6% with dark urine had G6PD deficiency.

## Conclusion

Approximately One in every 4 (38%) children aged 6months – 12years passing dark urine admitted SRRH had AKI. Having urine that is profoundly black or “coca-cola” coloured (urine colour grade ≥ *9 of the urine color grade chart)*, as well as having a reduced urine output were associated with development of AKI. Health workers in paediatric care should include reduced urine output as a 6th vital sign among children who present with history of passing dark urine.

Also, children who present in health facilities with profoundly black or coca-cola coloured urine and those who present with a reduced urine output should be targeted for screening for AKI, by carrying serial serum creatinine.

## Figures and Tables

**Figure 3:**
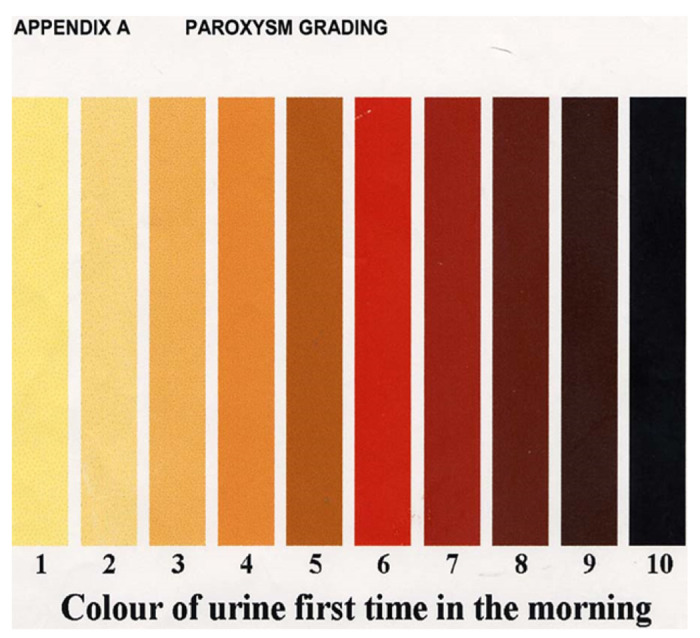
HammerSmith urine colour chart. Adopted from a study by Olupot-Olupot (1)

**Table 1:** Demographic and disease characteristics.

	Frequency (n)	Percentage (%)
Sex		
Male	157	61.6
Female	98	38.4
Weight, Mean (SD)	20.7(5.7)	
Height, Mean (SD)	119.4(17.0)	
Age, Mean (SD)	28**(0.7)**	
< 1year	1	0.4
1-5years	93	36.5
6-10years	128	50.2
>10years	33	12.9
Grade of urine on color chart at screening		
5	13	5.1
6	28	11.0
7	49	19.2
8	111	43.5
≥ 9	54	21.2
Episode of passing dark urine		
1st	103	40.6
2-5	104	40.9
6-10	41	16.1
>10	6	2.4
Reduced urine output	34	13.3
Early morning facial puffiness	20	7.8
Convulsions	16	6.3
Altered level of consciousness	11	4.3
Difficulty in breathing	14	5.5
Taken medications before coming	224	87.8
Antimalaria	189	84.4
Antibiotics **	52	20.4
Antipyretics/analgesic	202	79.2
Paracetamol	166	82.2
NSAIDS*	36	17.8
Received blood in this illness	94	36.9
Use of herbal medications in this illness	39	15.3

* NSAIDS include Diclofenac, Ibuprofen.

** Antibiotic include Gentamycin, Metronidazole, Amoxicillin, Xpen, Ceftriaxone

**Table 2:** Descriptive statistics of the participants’ laboratory results

Variable	Frequency(n=255)	Percentage (%)
Positive Blood slide for malaria	131	51.4
Parasitemia		
Malaria parasites 1+	60	45.8
Malaria parasites ≥ 2+	71	54.2
MRDT (Positive)	228	89.4
Haemoglobin concentration, Mean (SD)	6.72(2.86)	
Haematocrit	19.31(7.87)	
WBC Count, median (IQR)	10.65(6.8-17.9)	
Absolute Neutrophil count x 10^3^, median (IQR)	6.22(3.88-12)	
Platelet count, median (IQR)	171(120-253)	
Sickle cell test results		
HBAA	155	60.8
HBAS	30	11.8
HBSS	70	27.5
**Urine dipstick**		
Leukocytes		
Neg	156	81.7
+(25)	22	11.5
++(75)	8	4.2
+++(500)	5	2.6
Protein		
Neg	34	17.8
Trace	55	28.8
+(30)	53	27.8
++(100)	44	23.0
≥ +++(300)	5	2.6
**Blood (RBCs)**		
Hemolyzed		
Neg	26	14.1
+(10)	41	22.3
++(50)	68	37.0
+++(250)	49	26.6
Non Hemolyzed	4	2
**Urine microscopy**		
RBCs		
Not seen	192	75.3
Present	63	24.7

**Table 3:** Factors associated with AKI among children passing dark urine admitted at SRRH

Variable	AKI	No AKI	OR (95% CI)	P-Value	aOR (95% CI)	P-value
Altered level of consciousness						
Yes	8 (72.7)	3 (27.3)	4.64 (1.20-17.95)	**0.026**	**0.174 (0.02-1.72)**	0.134
No	89 (36.5)	155 (63.5)	1		**1**	
Difficult in breathing						
Yes	12 (85.7)	2 (14.3)	11.0 (2.41-50.35)	**0.002**	**5.737 (0.74-43.93)**	0.093
No	85 (35.3)	156 (64.7)	1			
Grade of urine colour						
Yes ≥ 9	30 (55.6)	24 (44.4)	2.5 (1.35-4.61)	**0.003**	**1**	
No <9	67 (33.3)	134 (66.7)	1		**3.004 (1.55-5.80)**	**0.001**
Reduced urine output						
Yes	26 (76.5)	8 (23.5)	6.87 (2.96-15.92)	**<0.001**	**3.432(1.16-10.13)**	**0.026**
No	71 (32.1)	150 (67.9)	1		**1**	
Early morning facial puffiness						
Yes	15 (75.0)	5 (25.0)	5.60 (1.96-15.95)	**0.001**	**3.244 (0.79-13.25)**	0.101
No	82 (34.9)	153 (65.1)	1		**1**	
Convulsions						
Yes	10 (62.5)	6 (37.5)	2.91 (1.02-8.28)	**0.045**	**2.136 (0.55-8.24)**	0.270
No	87 (36.4)	152 (63.6)	1		**1**	
Dehydration						
Yes	20 (45.5)	24 (54.5)	2.27 (1.17-4.38)	**0.015**	**1.322 (0.54-3.28)**	0.546
No	138 (65.4)	73 (34.6)	1		1	
Sex						
Female	38 (38.8)	98 (62.4)	1.06(0.62-1.76)	0.848	0.837 (0.46-1.53)	0.565
Male	59(37.6)	60 (61.2)	1		1	
Age						
< 5years	26 (43.3)	34 (56.7)	1		1	
≥ 5years	71(36.4)	124(63.6)	0.75 (0.41-1.35)	0.334	0.703 (0.35-1.37)	0.304
Malaria						
Yes	87 (38.2)	141 (61.8)	1		1	
No	10(39.6)	17 (63.0)	0.95 (0.41-1.92)	0.582	0.882(0.31-1.67)	0.660

## Data Availability

The original data set will be available by the corresponding author upon request.

## Abbreviations

AKI: Acute Kidney Injury
aOR: Adjusted odds ratios
ASLAM: African Society for Laboratory medicine
CFH: Cell-free Haemoglobin
CI: Confidence interval
GCP: Good Clinical Practice
GCLP: Good Clinical and Laboratory Practice
IOR: Interquartile range
KDIGO: Kidney Disease:Improving Global Outcome
LMIC: Low- and middle-Income countries
MRDT: Malaria Rapid diagnostic Test
NSAIDS: Non-Steroidal anti-inflammatory drugs
SRRH: Soroti Regional Referral Hospital
